# A Rare and Serious Syndrome That Requires Attention in Emergency Service: Traumatic Asphyxia

**DOI:** 10.1155/2015/359814

**Published:** 2015-05-24

**Authors:** Gultekin Gulbahar, Tevfik Kaplan, Ahmet Gokhan Gundogdu, Hatice Nurdan Baran, Burak Kazanci, Bulent Kocer, Serdar Han

**Affiliations:** ^1^Division of Thoracic Surgery, Dr. Nafiz Korez Sincan State Hospital, Ankara, Turkey; ^2^Department of Thoracic Surgery, Ufuk University School of Medicine, Ankara, Turkey; ^3^Division of Anesthesiology and Reanimation, Dr. Nafiz Korez Sincan State Hospital, Ankara, Turkey; ^4^Department of Neurosurgery, Ufuk University School of Medicine, Ankara, Turkey; ^5^Division of Thoracic Surgery, Ankara Numune Teaching and Research Hospital, Ankara, Turkey

## Abstract

Traumatic asphyxia is a rare syndrome caused by blunt thoracoabdominal trauma and characterized by cyanosis, edema, and subconjunctival and petechial hemorrhage on the face, neck, upper extremities, and the upper parts of the thorax. Traumatic asphyxia is usually diagnosed by history and inspection; however, the patient should be monitored more closely due to probable complications of thoracoabdominal injuries. Treatment is conservative, but the prognosis depends on the severity of the associated injuries. Herein we present a traumatic asphyxia due to an elevator accident in a 32-year-old male patient and discuss the diagnosis, treatment, and prognosis by reviewing the relevant literature.

## 1. Introduction

Traumatic asphyxia is a rare syndrome resulting from sudden, severe blunt trauma of the thorax and upper abdomen [1, 2]. It is characterized by cervicofacial cyanosis, edema, subconjunctival hemorrhage, and petechial eruptions on the face, neck, upper parts of the thoracic cage, and the upper extremities [3]. Commonly, thoracic injuries like pulmonary contusion, hemothorax, and pneumothorax accompany the situation [1, 2].

## 2. Case

**Figure 1:**
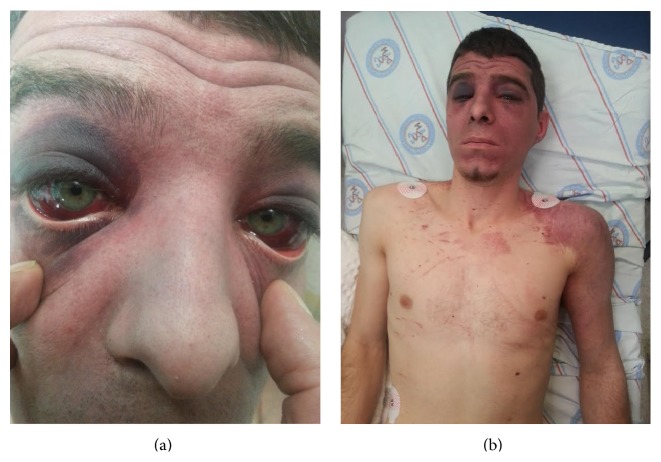
(a) The patient had bilateral subconjunctival hemorrhage. (b) The patient had facial cyanosis, petechial eruptions on the anterior surface of the thoracic cage and on left upper extremity.

**Figure 2:**
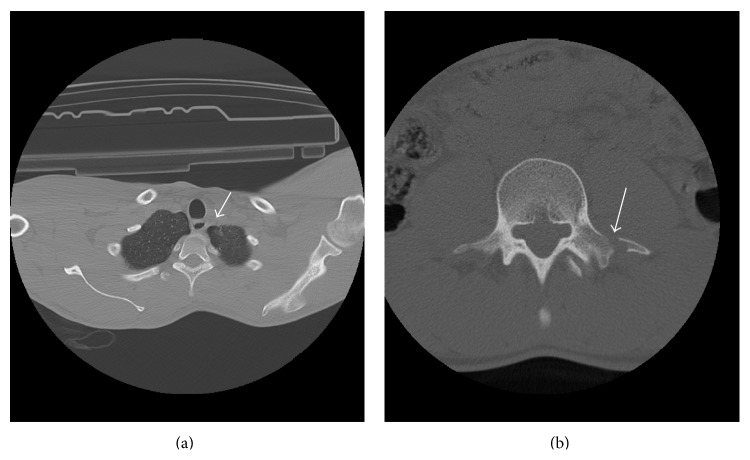
(a) Minimal pneumothorax was detected on computed axial tomography of the chest. (b) Fracture of the transverse processes of the first lumbar vertebrae was detected on computed axial tomography of the lumbar vertebrae.

**Figure 3:**
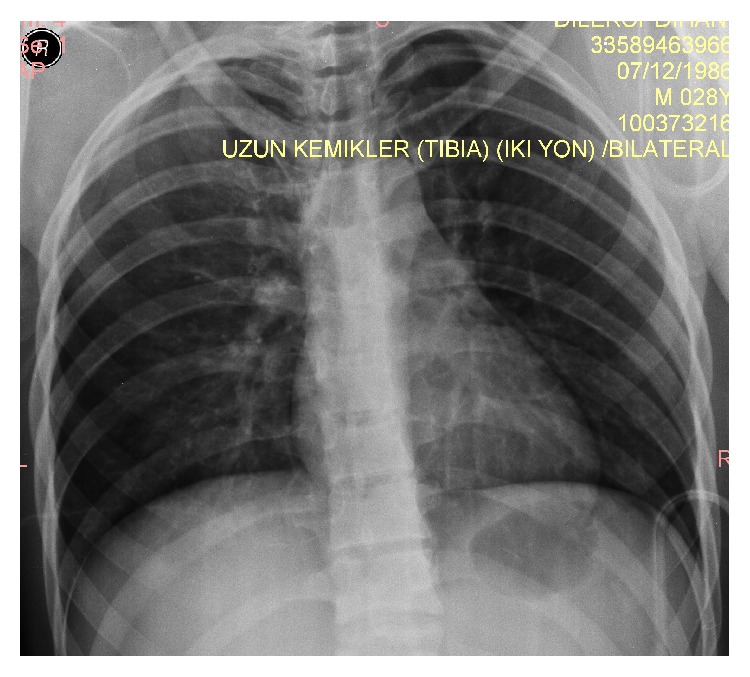
Chest X-ray of the patient.

A 32-year-old male patient was admitted to the emergency service after an industrial accident, in which an elevator cabin fell on him. The patient was conscious and had stable hemodynamic state on his initial examination. He had dyspnea, back pain, facial cyanosis, subconjunctival hemorrhage, and petechial eruptions on the anterior surface of the thoracic cage and on the left upper extremity (Figures 1(a) and 1(b)). Laboratory workup was consistent with the trauma. Bilateral pulmonary contusions, minimal pneumothorax, and fracture of the transverse processes of the first lumbar vertebrae were detected on computed axial tomography of the chest and lumbar vertebrae (Figures 2(a) and 2(b)). Chest X-ray examination of the patient revealed bilateral heterogeneous density increases (Figure 3).

The patient was hospitalized in the intensive care unit for follow-up. He was monitored in a continuous fashion. The head of the bed was elevated to 30 degrees to help overcome the increased intracranial pressure. Uninterrupted oxygen therapy was administered. Hemodynamic state of the patient, arterial blood gas analysis, oxygen saturation levels, and hematologic and biochemical parameters were checked at certain intervals. He was taken to the ward since no complication was observed on the first day of the trauma. The symptoms regressed and no complication was observed; thus he was discharged on the fifth day of hospitalization.

## 3. Discussion

Traumatic asphyxia is quite rare [3]. In 1937, when Oliver d'Angers first defined traumatic asphyxia, he used the term “ecchymotic mask” [4]. Traumatic cyanosis, compressive cyanosis, traumatic apnea, Oliver's syndrome, and Perth's syndrome have also been used to describe the situation [5, 6]. Usually, it follows a sudden and severe thoracoabdominal trauma, but it may also be seen in asthmatic attacks, epileptic seizure episodes, excessive vomiting, and cough cases [6, 7]. Sudden increase in the intrathoracic pressure is thought to be transmitted to the venous and capillary systems in head and neck, and these yields in stasis and rupture giving rise to the characteristic findings [3, 8]. Other possible mechanism is the lack of the valve system in superior vena cava, innominate vein, and jugular veins. Deeply situated structures drain into the internal jugular veins whereas the superficial ones drain into the external jugular system. Since the valves of the external jugular system cannot prevent the backflow, the symptoms are more common in the superficial structures like the scalp compared to the deeper structures like brain and the respiratory tract [9]. The compressed IVC protects the lower part of the body from injury during the course of the trauma.

The extent of the signs and symptoms depend on the duration and severity of the compression that thorax and upper abdomen are exposed to [10]. Many signs may accompany the characteristic findings. Confusion, amnesia, disorientation, uneasiness, agitation, hypoxia, cerebral edema, and hemorrhage are the most common ones [6]. Other than subconjunctival hemorrhage, decreased vision, blurred vision, papillary changes, optic nerve atrophy, diplopia, and exophthalmia are the most frequent ocular findings. Epistaxes due to capillary rupture, hearing loss due to the edema of the Eustachian tubes, or hemotympanum are the other probable findings [6, 8, 9]. Severe life-threatening conditions may coexist since the traumatic asphyxia develops as a result of major trauma to thorax, mediastinum, and upper abdomen. These are thoracic and extrathoracic injuries as pulmonary contusion, hemothorax, pneumothorax, flail chest, and hepatic laceration [1, 2, 9]. Signs of pulmonary injury like dyspnea, tachypnea, and hemoptysis may be observed. In rare cases, cardiac injury may be encountered [9]. An increase in the abdominal pressure may lead to organ injury, hematemesis, and hematuria. Apnea and hypoxemia associated with prolonged thoracic compression may be life threatening and give rise to increased mortality. The results may be such severe as shock, cerebral anoxia, neurological sequelae, and sudden death [7, 9].

Although traumatic asphyxia can be diagnosed easily through short anamnesis and physical examination, other reasons like superior vena cava syndrome and skull base fractures leading to subconjunctival hemorrhage and periorbital ecchymosis must be evaluated [7]. Careful and thorough examination is necessary to detect the thoracic and extrathoracic injuries that need urgent intervention. No severe injury other than bilateral pulmonary contusion and minor pneumothorax was found in our case.

Traumatic asphyxia cases must be monitored after securing the airway and fixing the cervical spine. Oxygen inhalation therapy and intravenous fluid replacement need to be initiated and the patient shall be intubated and followed on mechanical ventilation as needed [9]. The intubation may be difficult because of the airway edema, which is seen rarely [11]. Uncomplicated cases are treated conservatively. The head of the bed is preferred to be elevated at 30 degrees and oxygen administration is essential. This procedure decreases the intracranial pressure. Continuous monitorization of the patient, arterial blood gas, oxygen saturation level examinations at certain intervals, and supportive oxygen therapy are the key factors for the management throughout the hospitalization period [6, 10].

Traumatic asphyxia has almost perfect prognosis. It heals spontaneously within weeks except the neurological and ocular signs. In cases without accompanying injuries death may occur due to prolonged compression, apnea, and hypoxia [6, 10].

## 4. Conclusion

Traumatic asphyxia is a rare entity. Prognosis usually depends on the duration of the compression and the severity of the accompanying injuries. Quick and careful evaluation of the probable life-threatening conditions in the emergency service is crucial; however oxygen inhalation therapy, efficient ventilation, intravenous hydration, and measures to prevent the increase of intracranial pressure should be initiated as soon as possible.
